# A chip-integrated coherent photonic-phononic memory

**DOI:** 10.1038/s41467-017-00717-y

**Published:** 2017-09-18

**Authors:** Moritz Merklein, Birgit Stiller, Khu Vu, Stephen J. Madden, Benjamin J. Eggleton

**Affiliations:** 1 Centre for Ultrahigh Bandwidth Devices for Optical Systems (CUDOS), Institute of Photonics and Optical Science (IPOS), School of Physics, University of Sydney, Sydney, NSW 2006 Australia; 2 Australian Institute for Nanoscale Science and Technology (AINST), University of Sydney, Sydney, NSW 2006 Australia; 3 Centre for Ultrahigh Bandwidth Devices for Optical Systems (CUDOS), Laser Physics Centre, Research School of Physics and Engineering, Australian National University, Canberra, ACT 0200 Australia

## Abstract

Controlling and manipulating quanta of coherent acoustic vibrations—phonons—in integrated circuits has recently drawn a lot of attention, since phonons can function as unique links between radiofrequency and optical signals, allow access to quantum regimes and offer advanced signal processing capabilities. Recent approaches based on optomechanical resonators have achieved impressive quality factors allowing for storage of optical signals. However, so far these techniques have been limited in bandwidth and are incompatible with multi-wavelength operation. In this work, we experimentally demonstrate a coherent buffer in an integrated planar optical waveguide by transferring the optical information coherently to an acoustic hypersound wave. Optical information is extracted using the reverse process. These hypersound phonons have similar wavelengths as the optical photons but travel at five orders of magnitude lower velocity. We demonstrate the storage of phase and amplitude of optical information with gigahertz bandwidth and show operation at separate wavelengths with negligible cross-talk.

## Abstract (web summary)

Optical storage implementations based on optomechanical resonator are limited to one wavelength. Here, exploiting stimulated Brillouin scattering, the authors demonstrate a coherent optical memory based on a planar integrated waveguide, which can operate at different wavelengths without cross-talk.

## Introduction

Storing or delaying optical signals has been a major driving force for a wide variety of research efforts as it offers new possibilities in all-optical processing and enhanced light–matter interactions. An optical buffer that is able to maintain the coherence of the optial signal, i.e. storing amplitude and phase information, and is able to operate at multiple wavelengths would greatly enhance the capacity of photonic integrated circuits and optical interconnects. Coupling light to coherent acoustic phonons in optomechanical systems offers not only the opportunity to slow down the velocity of an optical pulse^1, 2^, but also enables a full transfer of an optical wave to an acoustic wave^3–6^, which subsequently can be transferred back to the optical domain after a certain storage time. Recent years have seen great progress in increasing the storage time in photonic–phononic whispering gallery mode resonators^4, 7–9^ and optomechanical cavities^10–12^, with reported storage times on the order of microseconds. Furthermore, the photon–phonon–photon transfer can be fully coherent^3, 11, 13^. However, there are several major challenges which need to be addressed before an optical memory based on this approach is compatible with all-optical information processing and transmission techniques.

First, any practical optical buffer needs, amongst other requirements, at least a gigahertz bandwidth. Previous demonstrations relied on structural resonances either in the form of high-Q resonators or suspended optomechanical cavities, in which the bandwidth is limited to sub-megahertz. There are several theoretical proposals to increase the bandwidth in optomechanical systems^14, 15^ but there has been no experimental demonstration to date.

Second, optical data transmission schemes usually harness multiple wavelength channels to increase the overall capacity. This means the storage process needs to work over a wide frequency range (a large number of channels); and it needs to preserve the frequency of the optical signal (no cross-talk between the channels). These requirements are challenging to fulfil in photonic–phononic systems relying on structural resonances, e.g. silica fibre-tip whispering gallery mode resonators or photonic–phononic crystal defect modes, since the optical wavelength is strictly bound to the resonance frequency. In the case of whispering gallery mode resonators, an optical pulse transferred to a phonon can be retrieved by a read pulse at a different wavelength^4^, so several wavelength channels cannot be stored and retrieved unambiguously, since the storage/retrieval process is not frequency preserving^16, 17^.

Finally, a practical optical buffer must be chip-integrable and able to be interfaced with other on-chip components, criteria not easily satisfied with other optomechanical platforms investigated to date. Fused silica fibre-tip resonators are micrometre size^4, 7–9, 13^ but cannot be easily implemented onto a planar chip platform. Lithographically produced photonic–phononic crystals, which form resonant cavities for the acoustic and optical modes, possess the requisite small footprint^10, 11^. Despite this, they either rely on fibre taper coupling^18, 19^ that can be challenging to operate outside a laboratory environment or require complicated under-etching processes to maximise the optical and acoustic Q-factor^20^. The under-etching step is required to confine the optical and acoustic modes in the out-of-plane direction and avoid leakage to the substrate, but limits compatibility with planar integrated photonic circuits.

Here, we demonstrate a different approach for coherent optical storage, harnessing travelling acoustic phonons in a planar integrated waveguide. We transfer the information carried by the optical signal to these acoustic phonons using stimulated Brillouin scattering (SBS)^21, 22^. We demonstrate that this transfer is fully coherent by storing and retrieving different phases. Our buffer does not rely on a structural resonance, so is not limited to a narrow bandwidth or single wavelength operation. We show that the unique phase-matching condition between travelling acoustic and optical waves allows the unambiguous storage and retrieval at several different wavelengths without cross-talk.

This result was enabled by a recent paradigm shift in SBS research from long lengths of optical fibre to chip-scale devices, allowing the excitation of coherent acoustic phonons on a chip using optical forces^23–27^. The opto-acoustic interaction strength is increased by several orders of magnitude by using carefully designed waveguides that guide optical as well as acoustic waves, allowing us to store broadband optical signals in a planar waveguide without relying on a resonator geometry. The acoustic phonons travel in the waveguide at a velocity that is five orders of magnitude slower than in the optical domain and do not suffer from effects of optical dispersion and other detrimental optical non-linearities during the delay process. Transferring the signal back to the optical domain leads to a delay of the optical signal by approximately the time the signal was encoded as acoustic wave.

In this article, we exploit this ultra-strong local opto-acoustic interaction in a highly nonlinear chalcogenide spiral waveguide to demonstrate storage of several optical bits with sub-ns pulse-width corresponding to a broad gigahertz bandwidth. We show the retrieval of the phase and amplitude information, multi-wavelength operation and continuously adjustable storage time over 21 pulse widths. We confirm our measurements using simulations based on coupled-mode equations showing excellent agreement with the experimental results. Our photonic–phononic memory operates at room temperature and only relies on a planar waveguide that can be interfaced with other on-chip components in a straight forward manner.

## Results

### Experimental approach

We use SBS—one of the strongest nonlinear effects—to coherently couple two optical waves and an acoustic wave. The optical data signal is transferred to the acoustic wave by a strong counter-propagating optical write signal. Using this nonlinear effect as a memory was first proposed in highly nonlinear fibre^22, 28^. The storage of only one amplitude level of several nano-second long pulses has been shown to date^22^. This is only a fraction of the capability this memory concept can offer.

**Fig. 1:**
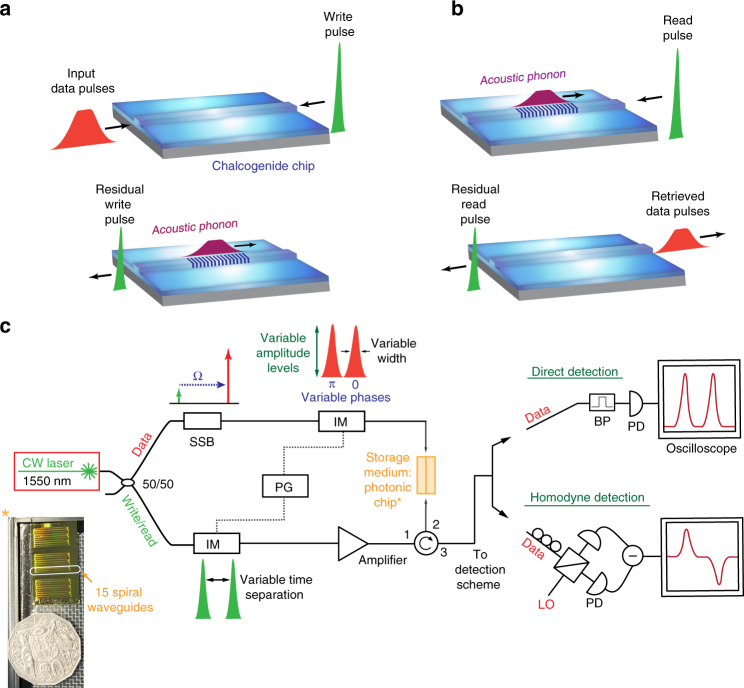
Basic principle and setup of the photonic–phononic memory. **a** Storing process: an optical data pulse is depleted by a strong counter-propagating write pulse, storing the data pulse as an acoustic phonon. **b** Retrieval process: in the retrieval process a read pulse depletes the acoustic wave, converting the data pulse back into the optical domain. **c** A basic schematic of the experimental setup. The inset shows a chalcogenide chip next to a 50-cent coin. The chip contains more than 100 spiral waveguides with different lengths (8.6, 11.7 and 23.7 cm). Note: this is only a schematic and the actual setup is more advanced and can be found in Supplementary Fig. 1 (CW continuous wave, SSB single-sideband modulator, IM intensity modulator, PG pulse generator, BP bandpass filter, PD photo-detector, LO local oscillator, Ω Brillouin frequency shift)

Our approach to write and retrieve the optical data pulses as acoustic phonons is schematically shown in Fig. 1a, b. A strong optical write pulse *ω*_write_, offset by the acoustic resonance frequency of the optical waveguide material, propagates counter to the optical data pulse *ω*_data_. When the two pulses encounter each other, the beat pattern between the two compresses the material periodically through a process known as electrostriction, exciting resonantly and locally a coherent acoustic phonon Ω = *ω*_data_ − *ω*_write_. The required power depends on the local Brillouin gain, which is orders of magnitude higher in chalcogenide As_2_S_3_ rib waveguides than, for example, in standard silica fibre. The optical and the acoustic modes are guided in the rib waveguide structure by the refractive index contrast and the acoustic impedance between the chalcogenide glass and the silica surrounding, respectively^29^. Once transferred to the acoustic wave the information on the optical data pulses can be retrieved after a storage time of several nanoseconds, corresponding to several tens of data pulse widths. The process is shown in Fig. 1b). A strong read pulse is coupled into the waveguide and retrieves the optical information by depleting the acoustic wave, the inverse process of the writing step. The setup for the photonic buffer is schematically shown in Fig. 1c) (a detailed description of the setup can be found in the ‘Methods’ section and in Supplementary Fig. 1).

**Fig. 2:**
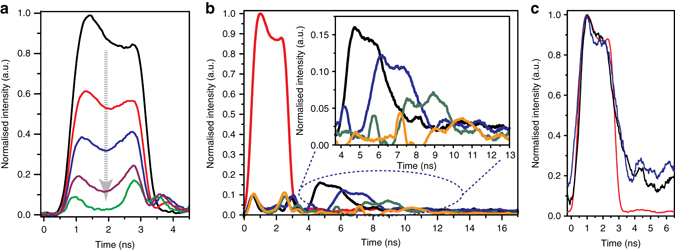
Store and retrieval process with tunable storage time. **a** Storing process: the optical data pulse is depleted by the counter-propagating write pulse, transferring the carried information to the acoustic phonon. The curves show the transmitted data pulse for different write pulse powers: *black*: original data, *red*: 1.1 W, *blue* 2.0 W, *purple*: 3.0 W, *green*: 4.7 W. Depletion of more than 90% can be achieved. **b** Retrieved data pulses after different storage times. The inset shows a zoomed-in version of the retrieved data pulses. **c** Original data pulse (*red*) super-imposed with the retrieved data pulses after 3.5 ns (*black*) and 5.5 ns (*blue*) respectively; the details of the original shape can be distinguished in the retrieved data pulses

### Photonic chip

As a storage medium we use a small footprint spiral waveguide made from the chalcogenide glass As_2_S_3_ comprising a rib waveguide structure with a cross-section of 2.2 μm by 800 nm. A photo of the chip is depicted in the inset of Fig. 1c) next to an Australian 50-cent coin. Every chip consists of spirals with several lengths ranging from around 9 to 24 cm. The spiral waveguides are grouped in quintets with a footprint per group of 20 × 0.7 mm. Longer waveguides are available by repeating the same spirals on one chip during the fabrication, leading to waveguides with up to 46 cm length. For details on the fabrication methods of the chip we refer to ref. ^30^. Lensed fibre-tips are used to couple light in and out of the waveguides. The chalcogenide glass is sandwiched between the silica substrate and the silica over-cladding. This not only provides guidance of the optical mode due to a contrast in the refractive index but provides also an acoustic impedance mismatch between the soft chalcogenide glass (*v*_sound_ = 2500 m/s) and the stiff silica (*v*_sound_ = 5996 m/s). Both the optical and the acoustic waves are guided in the chalcogenide glass, which provides a large opto-acoustic overlap. Ultra-high Brillouin gain of up to 50 dB amplification of a small continuous wave (CW) seed for a moderate CW pump power of 300 mW was achieved.

### Tunability of the storage time

**Fig. 3:**
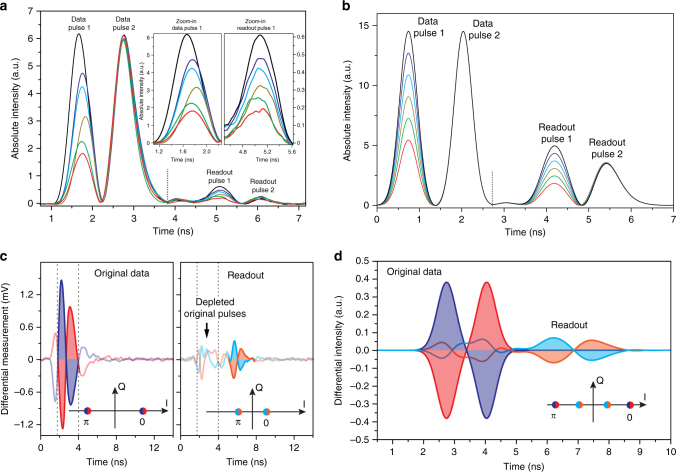
Amplitude and phase encoded signals. **a** Six different amplitude levels (depicted by the different colours) of a 500 ps optical pulse can be stored and retrieved after a storage time of 3.5 ns via direct detection. The amplitude of pulse 2 remains constant for the original and retrieved data pulse. The read-out efficiency of pulse 2 is lower due to practical limitations in the experiment (power limitation). The inset shows a zoom-in on the input amplitude levels and the readout amplitudes. **b** Simulation data of the amplitude response of the system. **c** Two phase levels of two 500 ps optical pulses, either 0 and *π* or *π* and 0 (depicted by the *red* and *blue* trace, respectively) are retrieved via homodyne detection after a storage time of 3.5 ns (*orange* and *light blue trace*). The insets show the two phases in phase space for the original (*red*/*blue*) and retrieved (*orange*/*light blue*) pulses. **d** Simulated phase response of the system (the colour schemes of the two encoded phases and the phase space diagram are according to the measurements depicted in **c**)

**Fig. 4:**
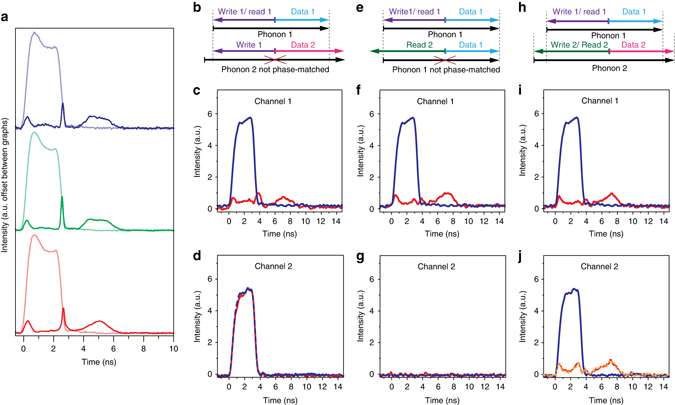
Multi-wavelength operation. **a** Multi-wavelength operation of the photonic–phononic memory for three different laser wavelengths *λ* for the data pulses (thin lines) (*blue curve*: 1549 nm, *green curve*: 1551.3 nm, *red curve*: 1552.9 nm). The efficiency of the memory remains the same (thick lines). The Brillouin frequency shift Ω was adjusted to 7.75 GHz, 7.74 GHz and 7.73 GHz, respectively. **b** Phase-matching condition for two data pulses and one write pulse phase-matched to data pulse 1 (corresponds to measurement **c**, **d**). The data pulse 2 is not phase-matched and therefore not affected by the write pulse 1. **c** Storage and retrieval of data pulse 1 (*blue* and *red curve*, respectively) while data pulse 2 (**d**) in a separate channel (100 GHz away from channel 1) is unaffected (*blue dashed line*: channel 1 off; *red line*: channel 1 stored and retrieved). **e** Phase-matching condition for two separate read pulses with data pulse 1 (corresponds to measurement **f**, **g**). One can see that read pulse 2 cannot readout phonon 1. **f** Shows the writing and retrieving of a data pulse (*blue* and *red curve*, respectively) in channel 1 while at the same time no readout pulse can be seen in channel 2 (**g**; *blue dashed line*: channel 1 off; *red line*: channel 1 stored and retrieved). **h** Phase-matching condition for two channels operating at two different wavelengths. **i** Storage and retrieval of data in channel 1 while simultaneously storing and retrieving data in channel 2 (**j**) (*blue* and *red curve*, respectively). For comparison the storage and retrieval of only channel 2 is depicted by the *dashed orange line* in **j**. Note: the difference in the noise floor between channel 1 and channel 2 is caused by the different noise properties of the two different photodiodes

The experimental realisations of an all-integrated multi-wavelength coherent photonic-phononic buffer is shown in Figs. 2, 3 and 4. Figure 2a shows the depletion of the optical data pulse with increasing counter-propagating write pulse power (storing process). For this experiment the storage medium is a 46 cm long spiral waveguide. Due to the ultra-strong Brillouin gain in chalcogenide waveguides, the depletion reaches over 90% with 20-fold lower write pulse peak power than in highly nonlinear fibre approaches^22^ for similar pulse conditions. The peak power levels of the interacting optical pulses presented in this article vary from 10 to 50 mW for the data pulses and 3 to 10 W for the write and read pulses depending on the overall gain of the individual waveguides.

The storage and subsequent retrieval of the optical data pulses are demonstrated in Fig. 2b). The storage time can be continuously adjusted by simply controlling the time difference between the read and the write pulses. A readout efficiency of 15–32% after 3.5 ns was achieved (see also Supplementary Note 3 and Supplementary Fig. 3). The inset of Fig. 2b shows a zoomed-in version of four examples of retrieved data pulses after different storage times. From the exponential decrease of the retrieval efficiency an acoustic decay time of 10.5 ns is measured using an exponential fit and is confirmed by a pump-probe measurement of the Brillouin gain linewidth (see Supplementary Note 2 and Supplementary Fig. 2). In order to study the retrieval of the pulse shape, we superimpose the normalised original data pulse with two normalised retrieved data pulses, displayed in Fig. 2c). The shape of the optical data pulse is maintained during the storage process indicating that the bandwidth of the photonic–phononic memory is large enough to resolve even small features, such as the peak at the beginning of the optical data pulse. The intrinsic Brillouin linewidth is only in the range of tens of megahertz, however due to the strong opto-acoustic coupling in the photonic–phononic waveguides the Brillouin response can be broadened to several gigahertz^31^.

### Phase coherence and multiple amplitude storage

We showed in the previous section that we can store nanosecond pulses in a waveguide with continuous tunable storage time while maintaining the pulse shape. In this section we show that we can extend the operational bandwidth of our memory much further, allowing the storage of sub-ns pulses with different amplitude levels. Furthermore we show that the transfer process of photon to phonon back to photon is fully coherent, enabling the storage of different phase states. These demonstrations show a significant increase in the capacity of the memory. The retrieval of the amplitude and phase information of two short optical pulses with 500 ps pulse width after 3.5 ns is shown in Fig. 3a, c. For the storage of these short pulses we used 24 cm long waveguides, hence a better signal-to-noise ratio (due to lower overall propagation loss) is achieved in comparison to the measurements presented in Fig. 2. The pulse width corresponds to a bandwidth of more than 1.5 GHz, almost two orders of magnitude wider than the intrinsic Brillouin linewidth. This implies a very high local Brillouin gain in the pulse overlap region as the Brillouin gain is spread out over a wide frequency range.

We encoded six different amplitude levels in pulse 1, while maintaining the amplitude level of a second data pulse constant as a reference. A comparison of the original and retrieved pulse 1 (inset) shows that we can easily distinguish six different amplitude levels; this can be enhanced with a more sensitive detection system. The amplitude of the second retrieved data pulse remains constant as does its original amplitude. We simulate our system using coupled-mode equations^32, 33^ and see great agreement with our measurements, presented in Fig. 3b) (more details on the simulation methods can be found in Supplementary Note 4).

Further to multiple amplitude levels, we can also store and retrieve different optical phases to show the coherence of the state transfer between travelling acoustic and optical waves. To distinguish the phase we replace the direct detection scheme (single photodiode) with an interferometric homodyne detection scheme. Here, the phase encoded signal interferes with a local oscillator and is detected by a balanced detector measuring the difference signal of two equal photodiodes.

Two pulses are encoded with two different phases, either 0 and *π* (*blue*) or *π* and 0 (*red*), respectively (Fig. 3c). After being stored for 3.5 ns, these same values can be read out (*light blue* and *orange*) and are clearly distinguishable. For a better understanding, the states in the phase space (I-Q diagram) are related to the optical pulses. For phase 0, the local oscillator and the data pulses interfere constructively, resulting in a positive value, for *π* they interfere destructively which results in a negative pulse on the balanced detector. The phase retrieval is possible due to the coherence of the Brillouin process and proves its potential as a coherent buffer. Note, that this feature can be implemented for any phase in the entire phase space and not only for 0 and *π*. Here, too, as for the amplitude measurements, we simulate our system and see excellent agreement between the measurements and the simulations (Fig. 3d).

### Multi-wavelength operation

Here, we demonstrate the multi-wavelength capabilities of our memory. On the one hand the memory operation must work at several different wavelengths, while on the other hand the cross-talk between wavelength channels should be minimal. Our Brillouin-based memory works at wavelengths where the waveguide is transparent, in contrast to resonator-based approaches where one is bound to the particular resonance frequencies. This transparency window reaches, in the case of chalcogenide, from the visible all the way to the deep infrared. To demonstrate the wavelength multiplexing capacity, we adjusted the operation laser wavelength to three different values in the tuning range of our laser. It can be seen from Fig. 4a that the same efficiency is achieved for all wavelengths. The pulse shape for the 1552.9 nm measurement is slightly distorted which can be assigned to the limitations in our setup (power limitations and effects of the nonlinear loop (Supplementary Note 1)) and is not of a fundamental nature. Every pair of frequencies (data frequency and read/write frequency) excites an acoustic wave at a specific frequency, which can be most easily seen in the equation for the Brillouin frequency shift Ω = 2*V*_A_ *n*_eff_/*λ* (Ω Brillouin frequency shift, *V*_A_ longitudinal acoustic velocity, *n*_eff_ effective refractive index and *λ* laser wavelength). The respective Brillouin shifts Ω are indicated in Fig. 4a.

The second important point concerns the cross-talk between different wavelength channels: here, the unique phase-matching conditions between travelling acoustic and optical waves inhibits mixing of different frequency channels (as illustrated in Fig. 4b, e, h). The storage and read-out process is strictly bound to specific phase-matching conditions, such that the process is operational at different wavelengths at the same time. To prove the point that there is no cross-talk between different channels we couple two data pulses, separated by only 100 GHz, simultaneously into the waveguide and measure the waveguide output using a dual channel oscilloscope (Fig. 4c, d). When adding write and read pulses phase-matched to the data pulses in channel 1 only the data pulse in this channel gets stored and retrieved (Fig. 4c) while there is no effect observable in channel 2 (Fig. 4d). This result shows that one can operate the memory on individual data streams, separated by a standard 100 GHz guard-band, without adding any detrimental distortions on the other channel.

We furthermore experimentally show that a non-phase-matched read pulse cannot retrieve information stored in a different frequency channel (Fig. 4f, g). To demonstrate this, we store and retrieve an optical data pulse in channel 1 (Fig. 4f), while simultaneously a second read pulse, separated by 100 GHz from the read pulse in channel 1, does not readout the stored data pulse, see Fig. 4g). This is a major difference to light storage schemes based on opto-mechanical resonator scheme where light interacts with standing acoustic waves or couples to transverse acoustic modes, as in these cases there is no or only a minimum momentum transfer. Therefore many different optical modes get modulated by the presence of the acoustic mode, hence these schemes are well-suited for wavelength conversion^17^.

Finally, we show that there is no cross-talk between the two wavelength channels separated by 100 GHz even when optical data pulses are stored and retrieved simultaneously in the two channels (Fig. 4i, j). For comparison, Fig. 4j shows also the stored and retrieved data pulse with the second channel turned off (*orange dashed line*).

## Discussion

In this article we have demonstrated a coherent photonic memory based on optically actuated travelling acoustic phonons in a planar waveguide. Our memory relies on a state transfer from photons to slowly propagating phonons and can therefore be seen as a completely different approach compared to schemes which rely on a reduced group velocity of light pulses such as coupled optical resonators^34–36^, photonic crystal cavities^37–39^ or slow-light schemes^38, 40–42^. By transferring the optical pulse to an acoustic wave, our optical buffer allows to circumvent detrimental optical dispersion effects^43, 44^ and allows for relatively long delay times of many pulse widths. The delay time can potentially be increased even further using a cascaded process^45^ or by further engineering the dissipation rate of the travelling acoustic phonon.

The photonic–phononic memory is fully controlled by the spatial–temporal overlap of the data, write and read optical pulses in a simple planar photonic circuit. Therefore the buffer is not an additional element of the circuit, but the photonic waveguide/link itself can be used as the buffering element bringing additional functionality to optical interconnects for next generation microelectronic networks^46–49^.

Storing and retrieving the full coherent information carried by the light signal enables the processing of multiple amplitude and phase levels, which is essential for contemporary communications schemes and greatly increases the number of bits that can be stored. The ultrahigh Brillouin gain in chalcogenide glass allows for the encoding of signals down to 500 ps pulse width. Even shorter pulses can be realized by further reducing waveguide losses and increasing the opto-acoustic coupling by tailoring acoustic properties. A very important feature of the demonstrated buffer is the operation at separate optical wavelengths without cross-talk. In particular the frequency preserving property due to the stringent phase-matching condition between travelling acoustic and optical waves is essential for multi-channel operation in order to store and retrieve information at different frequency channels unambiguously. In communication networks and computing architectures, this versatility plus the continuous tunability of the storage time of up to several nanoseconds enables precise and dynamic synchronization of optical data streams between several high-speed parallel processes.

## Methods

### Experimental setup for light storage

A narrow-linewidth distributed feedback laser at 1550 nm is divided into two arms—data and write and read arm—where the data pulse is frequency up-shifted by the Brillouin frequency shift Ω via a single-sideband modulator. The pulses are imprinted by two intensity modulators connected to a short-pulse generator. The write and read pulses are amplified by an erbium-doped fibre amplifier (EDFA). The amplified write and read pulses pass through a nonlinear fibre loop. The loop has two effects: firstly, it allows only the pulses to be transmitted and efficiently suppresses any noise or coherent background present from the laser or amplifier, respectively. Secondly, it improves the pulse shape by smoothing the edges of the pulses. After the loop a second EDFA amplifies the pulses again to reach the necessary peak power of several Watts. Bandpass filters (bandwidth 0.5 nm) are used in both arms to minimise the white noise from the EDFAs. Both paths lead to opposite sides of the photonic chip. The original and retrieved data pulses are observed by a 12 GHz photodiode connected to the oscilloscope. Before the photodiode a tunable narrowband filter is used to assure that only the data pulses reach the photodetector.

### Detection schemes

Two different detection schemes are used to detect the transmitted and retrieved data pulses: direct detection with a single photodiode is used for the amplitude retrieval whereas a homodyne detection scheme is used for the phase measurements. For the direct detection scheme a 12 GHz photodiode connected to the oscilloscope is used. For the homodyne detection scheme a local oscillator (CW) at the wavelength of the data pulses interferes at a 50:50 coupler with the original and retrieved data pulses. The beat signal is sent to a polarisation beam splitter both output signals of which are connected to a balanced photodetector. The polarisation of the local oscillator and the data pulses are controlled such that the difference signal of both photodiodes of the balanced photodetector is maximised in order to distinguish the two phases, shifted by *π*. In both detection schemes a tunable narrow-band filter (≈4 GHz) is used to assure that only the data pulses reach the photodetector.

### Data availability

The data that support the findings of this work are available from the corresponding author upon request.

## Electronic supplementary material

Supplementary Information
