# Author Correction: A chip-integrated coherent photonic-phononic memory

**DOI:** 10.1038/s41467-017-02506-z

**Published:** 2018-01-04

**Authors:** Moritz Merklein, Birgit Stiller, Khu Vu, Stephen J. Madden, Benjamin J. Eggleton

**Affiliations:** 1 Centre for Ultrahigh Bandwidth Devices for Optical Systems (CUDOS), Institute of Photonics and Optical Science (IPOS), School of Physics, University of Sydney, Sydney, NSW 2006 Australia; 2 Australian Institute for Nanoscale Science and Technology (AINST), University of Sydney, Sydney, NSW 2006 Australia; 3 Centre for Ultrahigh Bandwidth Devices for Optical Systems (CUDOS), Laser Physics Centre, Research School of Physics and Engineering, Australian National University, Canberra, ACT 0200 Australia

Correction to: *Nature Communications* 10.1038/s41467-017-00717-y, published online 18 September 2017

The original version of this Article omitted the Acknowledgements section:

“This work was sponsored by the Australian Research Council (ARC) Laureate Fellowship (FL120100029) and the Centre of Excellence program (CUDOS CE110001010). We acknowledge the support of the ANFF ACT.”

This has now been corrected in both the PDF and HTML versions of the Article.

