# Supplementary material for: A chip-integrated coherent photonic-phononic memory
Source: Nat Commun. 2017 Sep 18;8:574. doi: 10.1038/s41467-017-00717-y (PMC5603616; doi:10.1038/s41467-017-00717-y)
Supplement: Supplementary file 1 — Supplementary Information [file 41467_2017_717_MOESM1_ESM.pdf]

Supplementary Note 1: Detailed experimental setup. The experimental setup is shown in Supplementary Figure 1. A continuous wave (CW) narrow-linewidth distributed feedback (DFB) laser at 1550 nm is divided into a data and write-and-read arm by a 50/50 fibre coupler. The data pulses are frequency up-shifted by the Brillouin frequency shift $\Omega = 7.7$ GHz via a single-sideband modulator. The CW laser signal is carved into pulses by two intensity modulators connected to a short-pulse generator, allowing the generation of pulses with different amplitude levels and phase states. The pulses are amplified using erbium-doped fibre amplifiers (EDFA) and subsequently filtered by narrow bandwidth (0.5 nm) bandpass filters to reduce the effect of broadband white noise introduced by the amplification step. Additionally to the passive bandpass filter a nonlinear fibre loop is implemented in the write-and-read arm. The loop consists of 1 km standard single-mode fibre, a polarization controller and a 50/50 coupler to introduce some asymmetry in the two paths. This fibre loop is used for two reasons: firstly it allows only the pulses to be transmitted and efficiently suppresses any noise or coherent background present from the laser or amplifier respectively. Secondly, it improves the pulse shape by smoothing the edges of the pulses. After the loop a second EDFA amplifies the pulses again to reach the necessary peak power of several watts. Both paths lead to opposite sides of the photonic chip and are coupled to the waveguide using lensed fibres.

For the multi-wavelength measurement a second laser, 100 GHz apart from the first laser, is used and coupled into the data or write-and-read arm, respectively. The output from the chip (circulator port 3 in Supplementary Figure 1) is split with a 50 / 50 fibre coupler and sent to two

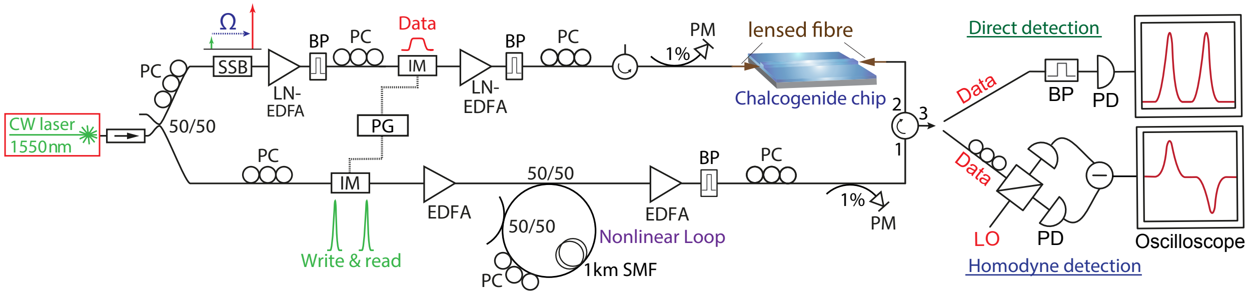

Supplementary Figure 1: **Experimental setup.** CW laser: continuous wave laser; PC: polarization controller; SSB: single sideband modulator; EDFA: erbium doped fibre amplifier; LN-EDFA: low-noise EDFA; BP: bandpass filter; SMF: standard single-mode fibre; PM: power meter; LO: local oscillator; PD: photodetector; Ω: Brillouin frequency shift.

narrowband filters to separate the two wavelength channels. Each filtered channel is then detected using two 12 GHz photodiodes connected to a dual channel oscilloscope.

Supplementary Note 2: Acoustic decay time. We analyzed the storage time of the phonon memory and compared it with a standard pump-probe measurement of the Brillouin linewidth. Supplementary Figure 2a) shows different readout pulses for different storage times up to 10.5 ns. For increasing storage times the readout efficiency decreases due to the decaying amplitude of the acoustic wave. The area of the retrieved pulses is integrated to determine the acoustic decay time (Supplementary Figure 2b). The exponential decrease of the pulse areas are plotted in Supplementary Figure 2c) and an exponential fit $\exp(-2t/\tau_{\mathrm{A}})$ reveals an acoustic decay time of 10.2 ns. We confirm the acoustic decay time by measuring the linewidth of the Brillouin gain response (Supplementary Figure 2c). A modified version of the setup in Supplementary Figure 1 is used to execute the CW pump-probe measurements. The intensity modulators were removed and the

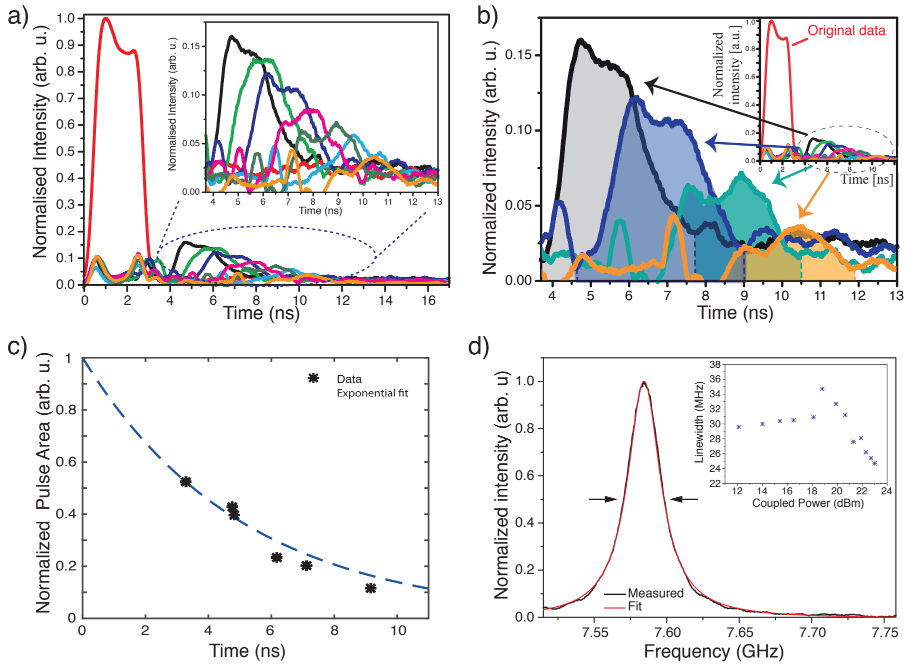

Supplementary Figure 2: **Storage time and acoustic decay time.** a) Measurements of different storage times indicated by the different colours of the curves. The inset shows a zoom-in of the retrieved pulses. b) Four retrieved data pulses with shaded colour filling indicating the pulse area used to determine the acoustic lifetime in (c). c) Pulse area with exponential fit $exp(-2t/\tau)$ reveals acoustic decay time of 10.2 ns. d) Brillouin linewidth measured with a CW pump-probe setup showing 30 MHz linewidth. The inset shows the linewidth for different pump powers.

single-sideband (SSB) modulator is frequency swept by the RF output of a vector network analyzer (VNA) to generate a seed signal. The transmitted seed signal is detected by a photodiode connected to the VNA and the Brillouin response for different pump powers is measured. The Brillouin gain linewidth ν_{B} relates to the acoustic decay time τ_{A} as $\tau_{\mathrm{A}} = 1/\pi\Delta\nu_{\mathrm{B}}$. The fit in Supplementary Figure 2c) shows a linewidth of 30 MHz for 21 dBm pump power, which agrees perfectly with the 10.2 ns decay time measured in the storage experiment. As expected the linewidth of the gain peak

decreases above the Brillouin threshold (inset Supplementary Figure 2d).

The great agreement of the two measurement techniques not only confirms the consistency of the light storage measurements, but also suggests itself for using the storage technique to locally access material and structure specific acoustic decay times. Whereas CW based pump-probe schemes only provide an average of the acoustic decay time over the whole length of the waveguide, the pulsed measurement determines the acoustic decay time at the point of the waveguide where the pulses overlap. The overlap can be scanned along the waveguide providing spatial information about the waveguide.

Supplementary Note 3: Maximum amplitude readout. The readout efficiency could be increased to 32% after 3.5 ns storage time as shown in Supplementary Figure 3. It is known from numerical studies that the amplitude of the retrieved pulses can be enhanced by using chirped pulses[1]. The 32% readout depicted in Supplementary Figure 3 is achieved by increasing the input power into the nonlinear loop (see setup Supplementary Figure 1), deliberately chirping the pulses to achieve the highest amplitude read-out efficiency. Achieving record readout amplitude efficiencies comes with distortions in the pulse shape. However, there are applications, where the overall readout amplitude is more important than the pulse shape, such as simple on-off keying schemes. In the same way one can increase the maximum retrieval time of the buffer by increasing the efficiency and therefore lifting the amplitude of the retrieved pulse above the noise floor. Higher input power increases the nonlinear process known as self phase modulation, chirping the pulses[2]. The nonlinear loop in the setup therefore not only reduces the noise, but also allows for a more

efficient readout amplitude through compression of the retrieved pulse. However the pulse shape is not maintained in this case. Besides using chirped pulses to improve the maximum retrieval ampli-

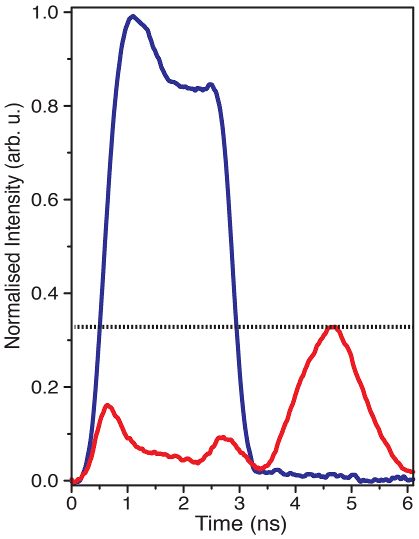

Supplementary Figure 3: **Maximising the amplitude retrieval efficiency.** A 32% retrieval efficiency of the pulse amplitude after a storage time of 3.5 ns was achieved (blue curve: original data; red curve: retrieved data).

tude, it was also shown theoretically that small amounts of chirp help to more efficiently excite the acoustic wave[3]. This can be understood by drawing an analogy to the McCall and Hahn area theorem for atomic two-level system[4]. Analogues to the π pulse in atomic resonances, a normalized pulse area of the write pulses can be defined and is given by [3] $\Theta = \sqrt{g_{\mathrm{B}} c / 8 A_{\mathrm{eff}} \tau_{\mathrm{B}} n} \times \int A(t) dt$, with the Brillouin gain coefficient g_{B}, the speed of light c, the effective mode area A_{eff}, the acoustic decay time τ_{B}, the refractive index n and the time integral over the pulse envelope $A(t)$. The maximum efficiency for exciting the acoustic wave is achieved when $\Theta = (m + 1/2)\pi$ with m being an integer number. However the data pulse cannot be transferred to the acoustic wave if the

pulse area is an integer multiple of π. If the pulse area is a multiple of π the first half of the pulse will write the acoustic wave, while the second half retrieves it again. However, for a linear chirped pulse the beginning of the pulse has a different frequency as the end of the pulse. Therefore, only a certain part of the pulse resonantly excites the acoustic wave and importantly does not de-excite the acoustic wave.

Supplementary Note 4: Simulation method. To simulate the phase and amplitude response of our system we solved standard coupled mode equations as presented in Ref. [1] using an implicit fourth order Runge-Kutta method[5]. The slowly-varying envelope coupled mode equations for a forward travelling pump wave A_{P}, a counterpropagating Stokes wave A_{S} and an acoustic wave Q can be written in the following form[1]:

$$\frac{\partial A_{\mathrm{P}}}{\partial z} + \frac{n}{c}\frac{\partial A_{\mathrm{P}}}{\partial t} = -\frac{g_0}{2A_{\mathrm{eff}}} Q A_{\mathrm{S}} - \frac{1}{2}\alpha A_{\mathrm{P}} \tag{1}$$

$$-\frac{\partial A_{\mathrm{S}}}{\partial z} + \frac{n}{c}\frac{\partial A_{\mathrm{S}}}{\partial t} = \frac{g_0}{2A_{\mathrm{eff}}} Q^* A_{\mathrm{P}} - \frac{1}{2}\alpha A_{\mathrm{S}} \tag{2}$$

$$2\tau_{\mathrm{B}}\frac{\partial Q}{\partial t} + Q = A_{\mathrm{P}} A_{\mathrm{P}}^* \tag{3}$$

The slowly varying envelopes A_{P}, A_{S} are normalized such that $|A_{\mathrm{P/S}}|^2$ is the power in watts, Q is the amplitude of the acoustic wave, n is the refractive index, c the speed of light, g_0 the Brillouin gain coefficient, A_{eff} the effective mode area, τ_{B} the acoustic lifetime and α the waveguide loss parameter.

The envelopes of the input data, write-and-read pulses are approximated to have Gaussian

form[3]:

$$A_{\mathrm{P/S}} = A_0 \exp(-\frac{1+iC}{2}\frac{t^2}{\tau^2}) \tag{4}$$

with the parameter C giving the chirp rate in GHz / ns following the definition of Ref. [3] and τ being the FWHM. The parameters used for the amplitude simulations (Fig. 3b) are as follows: n = 2.4, $g_0 = 0.715 \cdot 10^{-9}\mathrm{m/W}$, $A_{\mathrm{eff}} = 1.5 \cdot 10^{-15}\mathrm{m}^2$, $\tau_{\mathrm{B}} = 10.5\,\mathrm{ns}$, $\alpha = 0.2\,\mathrm{dB/cm}$. The FWHM of the data pulses is 500 ps and the peak power is varying in equidistant steps from 15 mW to 40 mW. The FWHM of the data pulses was 1 ns, the peak power 3.5 W and C = 0.88 GHz / ns. The temporal separation of the write and the read pulse was 3.5 ns. The parameters used for the phase simulations (Fig. 3d) are the same as for the amplitude simulations with 40 mW of data power and two different phases 0 and π.

Supplementary References

1. Winful, H. Chirped Brillouin dynamic gratings for storing and compressing light. *Optics Express* **21**, 10039–10047 (2013).
2. Boyd, R. W. *Nonlinear Optics* (Acad. Press, 2003).
3. Dong, M. & Winful, H. G. Area dependence of chirped-pulse stimulated Brillouin scattering: implications for stored light and dynamic gratings. *Journal of the Optical Society of America B* **32**, 2514-2519 (2015).
4. McCall, S. L. & Hahn, E. L. Self-Induced Transparency. *Physical Review* **183**, 457–485 (1969).
5. Sterke, C. M. D., Jackson, K. R. & Robert, B. D. Nonlinear coupled-mode equations on a finite interval: a numerical procedure. *Journal of the Optical Society of America B* **8**, 403–412 (1991).
